# Corticotropin-Releasing Factor Receptors in the Locus Coeruleus Modulate the Enhancement of Active Coping Behaviors Induced by Chronic Predator Odor Inoculation in Mice

**DOI:** 10.3389/fpsyg.2019.03028

**Published:** 2020-01-10

**Authors:** Qiong Wang, Yingjuan Liu, Jianxu Zhang, Weiwen Wang

**Affiliations:** ^1^School of Education, Zhengzhou University, Zhengzhou, China; ^2^School of Life Sciences and Technology, Nanyang Normal University, Nanyang, China; ^3^State Key Laboratory of Integrated Management of Pest Insects and Rodents in Agriculture, Institute of Zoology, Chinese Academy of Sciences, Beijing, China; ^4^CAS Key Laboratory of Mental Health, Institute of Psychology, Beijing, China; ^5^Department of Psychology, University of Chinese Academy of Sciences, Beijing, China

**Keywords:** stress inoculation, predator odor exposure, active coping, corticotropin-releasing factor receptors, locus coeruleus, dorsal raphe nuclei

## Abstract

Stress inoculation has been proved to induce active coping behaviors to subsequent stress. However, the specific neural mechanisms underlying this effect remain unclear. In this study, a chronic and mild predator odor exposure model was established to investigate the effect of predator odor stress inoculation on behaviors in novel predator odor exposure, open field test and forced swimming test (FST), and on the expression of CRF receptors in locus coeruleus (LC) and dorsal raphe nuclei (DRN). The results showed that predator odor stress inoculation increased the active coping of mice under the severe stress environment without changing the stress response to a new predator odor. Meanwhile, in LC, the CRFR1 expression was increased by predator odor stress inoculation. These results suggested that predator odor stress inoculation can be used as an effective training method to improve active response to later severe stress and the function of CRFR1 in LC might be a potential underlying biological mechanism.

## Introduction

Stress is a generalized set of physiological and psychological responses observed when an organism is placed under challenging circumstances (Greenberg et al., 2013; Wang et al., 2018). The adaptive and maladaptive responses to stressor are closely related to psychological health, and the intensity and duration of stress are important factors affecting the risk of mental disorders. Predator stress is a kind of instinctive response for maintaining the reproduction and survival of species (Philbert et al., 2015; St-Cyr and McGowan, 2015). The excessive predator stress has been documented to exert negative effects on behavior, emotion and cognition in the pattern of stress maladaptation (Apfelbach et al., 2005). For example, recent researches find that excessive predator stress may induce long-term anxiety and depressive behavior in rats (Lim et al., 2016; Wah et al., 2019; Wu et al., 2019). However, mild predator stress can lead to active coping behaviors in response to stress. Previous study has been reported that chronic exposure of mild cat odor enhanced aggression, urinary attractiveness, and sex pheromones of mice (Zhang et al., 2008). To increase the adaptive response to stress, stress inoculation may be an effective way.

Stress inoculation originally refers to mild but not minimal nor severe stress exposure early in life enhances subsequent coping (Lee et al., 2016). Many studies have confirmed the effects of stress inoculation. Abraham and Gruss find that stress experience in early life improves cognitive and emotional processing of stressors later in life in Octodon degus (Abraham and Gruss, 2010). In primate studies, researchers find that exposure to stress in early life reduces subsequent indications of anxiety, increases exploration of novel situations, and decreases cortisol levels after stress (Lyons et al., 2009; Lyons and Parker, 2010). Some human studies have also found that stress experience in childhood and adolescent can effectively improve the ability to cope with similar stressors in adulthood (Khoshaba and Maddi, 1999; Mortimer and Staff, 2004; Forest et al., 2010; Bosnjak et al., 2019; Navaee and Kaykha, 2019). These findings suggest that stress inoculation training may be a good method to enhance the adaptive response to stress, which is of great significance for the prevention and treatment of stress-related mental diseases.

As the core neurohormone to initiate the stress response, corticotropin-releasing factor (CRF) has extensive neural connections with monoamine transmitters system (Henckens et al., 2016; Spierling et al., 2017), which is the most important stress system in brain, and plays an important role in stress response and coping strategies, but how are these systems involved in the process of stress inoculation is not clear. Many investigations indicate that the dorsal raphe nuclei (DRN) 5-HT system is compelling as a target of CRF given the established role of this system in stress coping (Lesch, 2001; Schindler et al., 2012). Two CRF receptor subtypes, CRF-R1 and CRF-R2, are distributed in DRN, these two receptors regulate the active and passive stress coping by a bimodal regulation of DRN-5-HT neuronal activity. Selective blockade CRF-R2, but not CRF-R1, decrease the passive responses induced by inescapable shock and swim stress (Hammack et al., 2003; Valentino et al., 2010). On the contrary, administration of CRF-R1 agonist into the DRN that inhibit the DRN-5-HT system and prevent learned helplessness produced by inescapable shock and swim stress, as well as facilitate an active escape response (Hammack et al., 2003, 2012; Valentino et al., 2010).

The locus coeruleus (LC), an important target of CRF neuronal projection, is critically involved in stress response and stress coping. CRF directly mediates the activation of LC- norepinephrine (NE) system during stress, and the CRF-R1 (only type of CRF receptor in LC) plays an important role in regulation of norepinephrine nervous system (Sánchez et al., 1999). Administration of CRF increases noradrenergic neurons activity and promotes NE release in LC (Curtis et al., 2012; Lavicky and Dunn, 2010; Page and Abercrombie, 2015). In forced swimming test (FST) the passive response is decreased by partial denervation of the LC-NE activity (Häidkind et al., 2003). In addition, antagonism of CRF receptors in the LC region is effective in attenuating immobilization stress induced defensive withdrawal in rats (Smagin et al., 1996). CRF micro-injection into LC increases an adaptive mechanism in an attentional set-shifting task with an inverted U-shaped dose-effect relationship (Snyder et al., 2012). These studies indicate that CFR in LC may also be involved in the regulation of coping strategies in stress.

The present experiments were designed to explore the stress inoculation effect of mild predator stress on the stress coping strategy, and its neurobiological mechanism was also discussed by investigated the expression of CRF receptor in LC and DRN. Firstly, the predator–prey interaction system between rats and mice was used to build an animal model of mild predator stress inoculation. Secondly, we tested the stress responses of animals in a similar predator stress, and whether there was a wider stress inoculation effect in a similar non-predator but threat stress condition. Furthermore, we attempted to explore the neurobiological foundation of predator stress induced stress inoculation effect by investigated the expression characteristics of CRF receptors in LC and DRN.

## Materials and Methods

### Animals

Twenty-six male ICR mice were purchased from Weitong-Lihua Laboratory Animal Company, Beijing, China. They were individually kept in plastic cages (27 cm × 12 cm × 17 cm) under the 14:10 h (light: dark) light regime (light on at 6:00 PM) and at the temperature of 21 ± 0.2°C. Food (standard mouse chow) and water were provided *ad libitum*. They were used for experiments after 4 weeks of acclimation.

At the beginning of the experiment, the mice were randomly assigned to one of two groups, each with 13 individuals. These were housed in two separate rooms for testing the effects of exposure to rat urine and rabbit urine, respectively. The mice of the two groups did not differ in body weight at the beginning of the experiment. All experimental procedures were performed with the approval of the Institutional Review Board of the Institute of Psychology at the Chinese Academy of Sciences and according to the guidelines of the National Institutes of Health Guide for the Care and Use of Laboratory Animals (NIH Publication number 80-23).

### Urine Collection

The urine was collected as described before (Zhang et al., 2008). Rat urine for use as a predator odor was collected. We collected rabbit (Oryctolagus cuniculus) urine as a non-predator novel odor from four adult males (New Zealand white strain, raised on Rabbit Chow 5326 Laboratory Diet) individually housed in the animal unit under the 12:12 h (light: dark) light regime. Urine was collected by placing each rat or rabbit individually in a clean cage with a grid floor and setting a clean collecting pan (with plastic films) underneath. After the animals had urinated into the collecting pan, the urine was immediately collected into vials and placed in a freezer for storage at –20°C. Potential contaminated urine, such as that deposited with or next to feces, was rejected. We pooled urine samples from conspecific individuals and used these for treating the experimental subjects.

### Predator Odor Exposure

To prepare the odor samples to be presented to test subjects, we used a micro-syringe to inject 5 μl rat urine, rabbit urine into a glass capillary (ID 1.5 mm, OD 2.0 mm, length 10 cm), which was then sealed with Bio-seal at one end. The samples stayed inside the micropipette in such a way that test subjects could not come into contact with the urine even if they occasionally touched the capillary. We hung the capillary at the side panel of each feeder, above the lid and just beyond the reach of the mice. The snout of the mouse could come no nearer than 1 cm. We renewed the urine in the capillary daily to keep the odor stimulus fresh for 31 consecutive days as stress inoculation training. After that, a set of behavioral tests were performed as follows.

### Behavior Tests

#### Novel Odor Exposure

The novel predator odor exposure was designed to test whether early predator odor exposure (rat urine) induced habituation to the predator odor (ferret urine) in later life. For the novel odor exposure test, we provided the cattle urine samples and ferret urine samples as novel odor to rat urine-exposed mice and rabbit urine-exposed mice, respectively. We allowed 2 min between trials. The time spent in sniffing (within 1 cm of the rod) and licking each rod tip using stopwatches were measured. To control for experimenter bias, the experimenter was blind to the nature of the sample.

#### Open Field (OF) Test

Twenty-four hours after the novel odor exposure, the mice were tested in the open field. The open field test was designed to test anxiety-like behavior and active coping behavior (exploration) in mild stressful condition induced by early predator odor exposure. The apparatus was a plexiglas open field box (50 × 50 × 30 cm), the floor was divided into 25 equal squares, the three squares in the center of the open field were center grid, others were periphery grid. On each test, the mouse was placed in the corner of the open field, the observation lasted for 10 min. The latency of the mice left the corner where it was put in, the center grids and periphery grids which the mice crossed, the amount of time spent by each animal in either center grid or periphery grid, defecation score, urine score, rearing, and grooming were counted. Between each test in the interval, the box was cleaned by alcohol and then water.

#### Forced Swimming Test (FST)

The forced swimming test was designed to inspect whether the early stress experience (predator odor exposure) triggered the emergence of active coping behaviors in more intense stressful condition in later life. The protocol was adapted from previous studies (Xia et al., 2007). One day after the open field, the mice were tested in FST. At the beginning of the test, each subject was dropped into glass cylinders (height, 25 cm; diameter, 10 cm) containing 10 cm water, maintained at 24 ± 1°C. All animals were forced to swim for 6 min, each session was recorded by a video camera and the duration of test was measured during the final 4 min of the 6 min test. The immobility was defined that the mouse floating in the water without struggling and making only those movements necessary to keep its head above the water. The duration of immobility, swimming and climbing was recorded in this test.

### Immunohistochemistry (IHC)

The IHC employed here was similar to that described in previous studies (Dermitzaki et al., 2007; Meng et al., 2011). After the behavior tests, mice were executed and brains was removed and routinely fixed in 0.01M PBS containing 4% paraformaldehyde (PFA), areas of interest (−3.08 to −7.08 mm from bregma for dorsal raphe nucleus and LC) were dissected and then post-fixed in 0.01M PBS containing 4% PFA for 6 h. Afterward the specimens were rinsed with 0.01M PBS (5 × 30 min, 4°C) and were dehydrated as follows: 3 × 30 min 70%, 90% and 96% ethanol, 3 × 30 min 100% ethanol and 3 × 30 min Roti-Histol. Brain samples were pre-embedded for 3 × 12 h in different Roti-Histol/paraffin solutions (2:1, 1:1, and 1:2) and 3 × 24 h in pure paraffin at 58°C. Then the samples were embedded in paraffin and mounted on standard cassettes and coronal paraffin sections (4 μm) were cut with a microtome (Leica 235) at room temperature, transferred onto coated slides (2 sections per slide) and dried on a heating plate at 58°C for 30 min.

The sections were decorated 3 × 5 min in Roti-Histol followed by 2 × 5 min in 100% ethanol and 5 min in 96%, 90%, and 70% ethanol. They were then washed 3 × 2 min in 0.05M PBS at room temperature. The sections were heated in a citrate buffer solution in a microwave oven. The slides were then washed in 0.05 M PBS (3 × 2 min) at room temperature, incubated in a blocking solution (3% hydrogen peroxide) for 10 min, and subsequently washed again (3 × 2 min) in 0.05M TBS at room temperature and incubated in a blocking solution (0.01M PBS containing 10% normal bovine serum and 1% BSA) for 20 min at room temperature. Afterward, the sections were incubated with anti-CRFR1 (sc-12381; 1:50, Santa Cruz Biotechnology) and anti-CRFR2 (sc-20550; 1:50, Santa Cruz Biotechnology) goat polyclonal IgG overnight at 4°C, washed in 0.05M PBS (3 × 5 min) at room temperature, then incubated in the Polink-2 plus Polymer HRP detection System (PV-9000) from Beijing Zhongshan Biotechnology Co. (Beijing, China). Finally, after washing in 0.05M PBS (3 × 5 min) at room temperature, slide-mounted brain sections were immunoreacted with 0.003% hydrogen peroxide in the presence of 0.05% 3,3′-dianino-benzidine (DAB). The slides were then dehydrated by serial alcohol rinsing as follows: 3 × 30 min 70%, 90%, and 96% ethanol, 3 × 30 min 100% ethanol, dewaxed in dimethylbenzene, and cover-slipped in the histofluid mountant.

### Quantification and Statistical Analyses

The number of CRFR1/CRFR2 positive cells were counted using a light microscope (Olympus BX-51 with Camedia Master C-3040 digital camera) and image analysis software (Image-pro plus 6.0). For analysis, cell counts were averaged into a single score for each mouse. Results were presented as mean ± standard error for all measures. The group difference of behavior tests and LC and DRN CRFR1/CRFR2 expression in IHC between rat urine exposure group and rabbit urine exposure group were examined by Student’s *t*-test using SPSS 13.0 software. The significance level was defined as *p* < 0.05.

## Results

### The Effects of Predator Odor Exposure on Behaviors in Novel Odor Exposure Test

**FIGURE 1:**
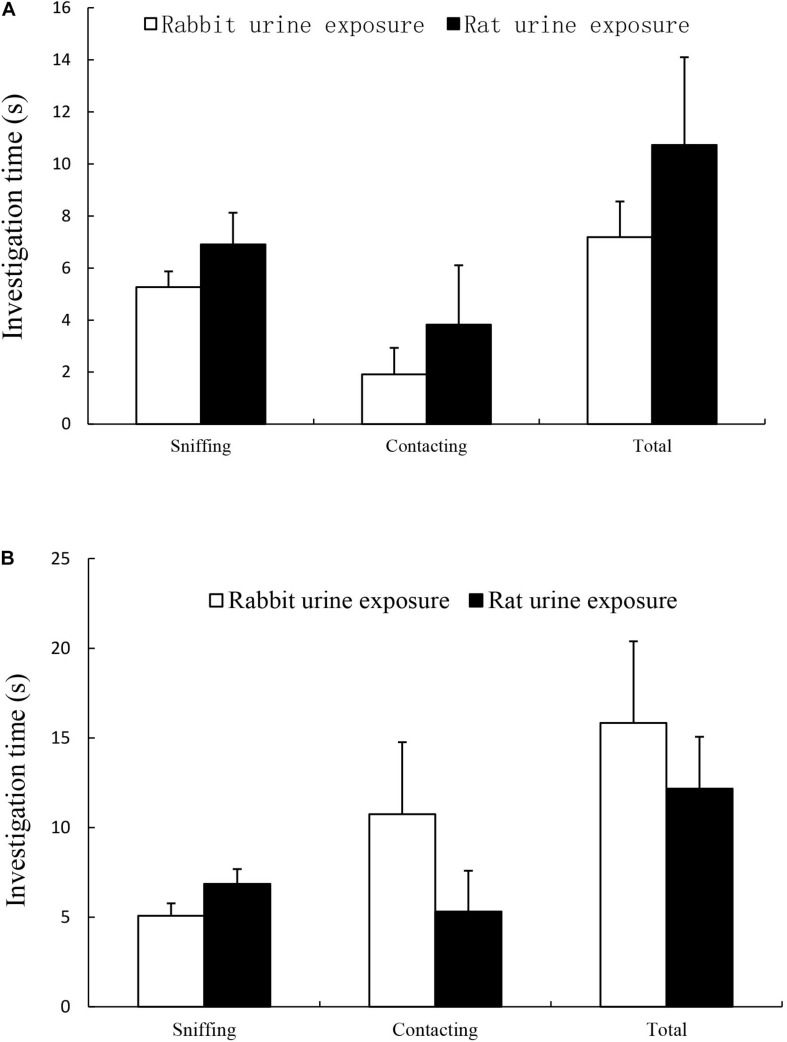
Time spent on sniffing, licking and total (sniffing + licking) in rabbit urine exposure group and rat urine exposure group, including exposure to cattle urine samples **(A)** and ferret urine samples **(B)**. Data are expressed as the mean ± SEM.

The results were summarized in Figure 1, there was no difference in time spent in sniffing and licking rod tip within cattle urine samples (Figure 1A) or ferret urine samples (Figure 1B) between rat urine exposure group and rabbit urine exposure group.

### The Effects of Predator Odor Exposure on Behaviors in Open Field Test

The results of open field test are showed in Table 1, predator odor exposure marginally significantly decreased the amount of time spent in either center grid (*t*_25_ = 1.983, *p* = 0.059). There were no changes in other behaviors in open field test between rat urine exposure group and rabbit urine exposure group mice.

**TABLE 1:** Effects of predator odor exposure on behaviors in open field test.

**Behavior pattern**	**Rabbit urine exposure mice**	**Rat urine exposure mice**
Latency	11.77 ± 3.053	10.38 ± 1.704
Center grid	52.08 ± 4.972	49.23 ± 3.703
Periphery grid	214.5 ± 19.40	247.8 ± 13.53
Locomotion	266.5 ± 21.83	297.0 ± 15.85
Center grid/Locomotion	0.199 ± 0.014	0.166 ± 0.009^∗^
Center time	68.91 ± 5.791	56.54 ± 6.166
Climbing	81.69 ± 5.481	86.38 ± 3.990
Rearing	42.23 ± 6.786	37.62 ± 5.677
Total	123.9 ± 9.423	124.0 ± 6.293
Grooming num	4.692 ± 0.702	3.462 ± 0.489
Grooming time	11.57 ± 1.878	8.632 ± 1.292
Urination	4.154 ± 0.750	2.923 ± 0.655
Defecation	6.462 ± 0.789	6.154 ± 0.807

*^∗^*p* = 0.059, compared to rabbit urine exposure mice.*

### The Effects of Predator Odor Exposure on Behaviors in Forced Swimming Test

**FIGURE 2:**
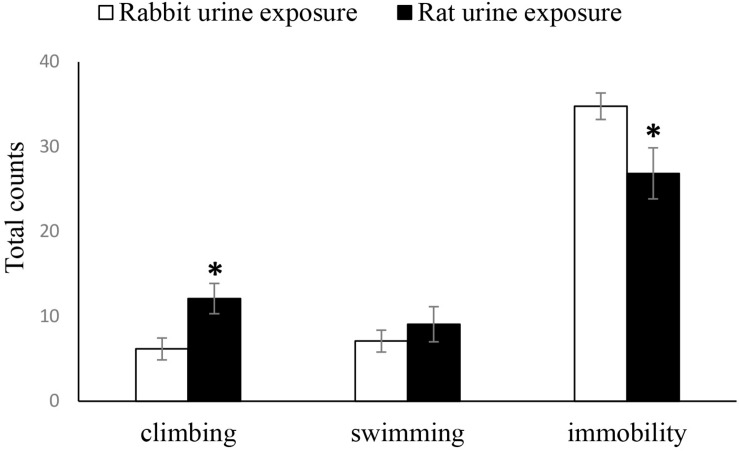
Duration of immobility, swimming and climbing in rabbit urine exposure group, and rat urine exposure group. Data are expressed as the mean ± SEM (^∗^*p* < 0.05 compared with the rabbit urine exposure group).

As shown in Figure 2, the duration of immobility time in the FST of the rabbit urine exposure group mice was significantly longer than that of the rat urine exposure group mice (*t*_25_ = 2.339, *p* = 0.028), inversely, there was a significantly increased duration of climbing time of the rat urine exposure group than that of the rabbit urine exposure group mice (*t*_25_ = −2.697, *p* = 0.013), and the duration of swimming time was not differ between the rat urine exposure group and rabbit urine exposure group mice.

### The Effects of Predator Odor Exposure on Corticotropin-Releasing Factor Receptors Expression in Locus Coeruleus and Dorsal Raphe Nuclei

**FIGURE 3:**
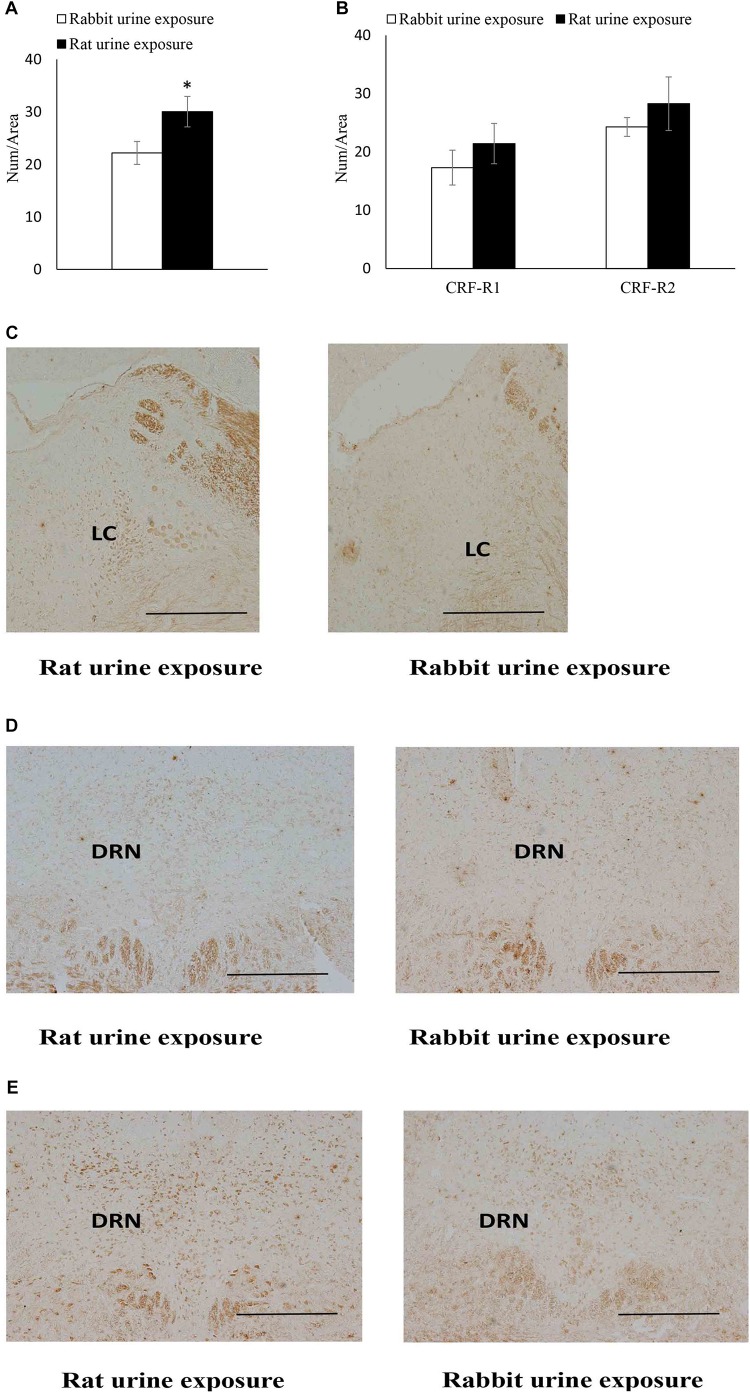
Expression of CRF receptors in rabbit urine exposure group and rat urine exposure group, including CRFR1 expression in locus coeruleus **(A)** CRFR1, CRFR2 expression in dorsal raphe nucleus **(B)**, representative immunohistochemistry (IHC) figures of CRFR1 expression in LC **(C)** CRFR1, **(D)** CRFR2, and **(E)** expression in DRN. Data are expressed as the mean ± SEM (^∗^*p* < 0.05 compared with the rabbit urine exposure group, Scale bar = 250 μm).

The expression of CRF receptors in each group was summarized in Figure 3. The rat urine exposure resulted in a significantly increase in the CRFR1 protein expression in the LC (*t*_22_ = 2.23, *p* = 0.039) compared with rabbit urine exposure group (Figure 3A). In the dorsal raphe nucleus, the rat urine exposure resulted in no significantly difference CRFR1 and CRFR2 protein expression compared of rabbit urine exposure group (Figure 3B).

## Discussion

In order to verify the effect of predator odor stress inoculation and explore its potential neurobiological mechanism, the current study examined the effects of predator odor exposure both on the behaviors and CRF receptor expression in DRN and LC. The results of present study showed that the responses to cattle and ferret urine were not significantly different between rat urine exposed mice and rabbit urine exposed mice in the novel odor exposure test. In addition, predator odor exposure didn’t change the locomotor activity but moderately increased the anxiety-like behavior of mice in open field test. In FST, predator odor exposure significantly increased the duration of climbing and decreased the duration of immobility. The results of immunohistochemistry showed that the CRFR1 protein expression in the LC was significantly increased by predator odor exposure, but the expression of CRFR1 and CRFR2 in DRN was not effected by predator odor stress inoculation. These results suggested that predator odor stress inoculation can improve active response to subsequent severe stress and the function of CRFR1 in LC might be involved in this effect.

Firstly, the responses to ferret urine between rat urine exposed mice and rabbit urine exposed mice were not significantly different in the novel odor exposure, suggested that early predator odor exposure did not induce habituation to novel predator odor. The novel odor exposure was described in previous studies (Zhang et al., 2008; Zhang and Zhang, 2010), different predator and non-predator odors (ferret urine and cattle urine) were used for avoiding the habituation to the same odor used in predator odor exposure. In addition, exposure to rat urine moderately reduced the ratio of center grid and locomotion in open field test, which means that predator stress increased anxiety-like behavior in mice. Consistently, previous study has reported that rats exposed to a brief cat odor showed anxiogenic responses and decreased exploration in the hole board test (Zangrossi and File, 1992). Chronic exposure to rat odor significantly increased anxiety-like behaviors in mice (Calvo–Torrent et al., 1999). These studies support the results of the present study. However, Adamec et al. (2010) found that predator stress decreased anxiety-like behavior in the light/dark box test and risk assessment in the EPM, CRF-R1 antagonism blocked initiation and consolidation of predator stressor effects on anxiety, and decreased risk assessment in the EPM. These different results may attribute to the different kind of predator stressors and the different behavioral experimental paradigms.

Secondly, the present results also found that exposure to rat urine decreased the duration of immobility and increased the duration of climbing in FST, suggested that predator odor stress inoculation increased the active coping and decreased the passive coping in later stress. Previous research has also shown that rats exposed to predator stress in adolescent mice showed significantly reduced immobility in the FST in early adulthood (Kendig et al., 2011). Another study also found that chronic exposure of predator stress in juvenile decreased the immobile times of Wistar Kyoto rats in adulthood in FST (Chen et al., 2014). These results are consistent with the findings of the present study. Taking together, these findings suggested that mild predator stress (rat urine) did not induce habituation to the predator stress later in life, indicated that the survival strategy of mice was intact. But the early stress experience triggered the emergence of active coping behaviors in more intense stressful condition (FST) later in life.

Thirdly, the present data indicated that predator odor exposure only significantly increased the CRF-R1 protein expression in LC, but the expression of CRF-R1 and CRF-R2 in DRN was not observably changed. These results suggested that LC CRF, rather than DRN CRF, involved in the enhancement of active coping induced by predator odor stress inoculation. Pobbeab et al. (2011) reported that selectively blocked 5-HT in DRN did not change behavioral responses of mice confronted with a predator, which supported the results of the present study. Previous study reported that predator stressor increased tonic LC discharge and decreased phasic auditory-evoked discharge, and this stress-induced alteration in LC discharge toward a high tonic mode was prevented by a CRF antagonist (Curtis et al., 2012). In addition to predator stress, researchers also found that repeated social stress decreases LC activity and CRF-R1 expression in LC (Chaijale et al., 2013). These studies have shown that the influence of CRF-R1 on LC neural activity played an important role in stress response, the mechanisms underlying this may explain how previous stress experience promoted the active coping behaviors. One mechanism through which this can occur was stress-induced CRF receptor redistribution, which was associated with changes in LC neuronal sensitivity to CRF (Reyes et al., 2008).

## Conclusion

In summary, using the experimental paradigm of predator odor stress inoculation, evidences were provided that stress inoculation enhanced the sensitivity of the acute response system to stress. These changes were characterized by increasing the active coping strategy in severe stress condition. Meanwhile, the survival strategy of mice under the threat of predator was not affected. The neuro-modulation of CRF system in LC rather than in DRN constituted the potential neurobiological mechanism of this effect. These results reminded us that stress inoculation could be used as a training method to improve the adaptive response to secondary stress.

## Data Availability Statement

The raw data supporting the conclusions of this manuscript will be made available by the authors, without undue reservation, to any qualified researcher.

## Ethics Statement

The animal study was reviewed and approved by the Institutional Review Board of the Institute of Psychology at the Chinese Academy of Sciences.

## Author Contributions

WW and JZ designed the research. QW and YL performed the research and acquired the data. QW, YL, WW, and JZ interpreted and analyzed the data. QW and WW drafted, revised, and wrote the manuscript.

## Conflict of Interest

The authors declare that the research was conducted in the absence of any commercial or financial relationships that could be construed as a potential conflict of interest.
